# Soluble Fraction from Lysates of Selected Probiotic Strains Differently Influences Re-Epithelialization of HaCaT Scratched Monolayer Through a Mechanism Involving Nitric Oxide Synthase 2

**DOI:** 10.3390/biom9120756

**Published:** 2019-11-21

**Authors:** Francesca Lombardi, Paola Palumbo, Antonella Mattei, Francesca Rosaria Augello, Maria Grazia Cifone, Maurizio Giuliani, Benedetta Cinque

**Affiliations:** 1 Department of Life, Health & Environmental Sciences, University of L’Aquila, Building Delta 6, Coppito, 67100 L’Aquila, Italy; francesca.lombardi@univaq.it (F.L.); paola.palumbo@univaq.it (P.P.); antonella.mattei@univaq.it (A.M.); francescaaugello@gmail.com (F.R.A.); mariagrazia.cifone@univaq.it (M.G.C.); maurizio.giuliani@univaq.it (M.G.); 2 Unit of Plastic and Reconstructive Surgery, Casa di Cura “Di Lorenzo” SrL, Via Vittorio Veneto 37, Avezzano, 67051 L’Aquila, Italy

**Keywords:** probiotics, wound healing, NOS2, NOS2 inhibitor, HaCaT

## Abstract

A growing body of evidence supports the use of probiotics in the treatment of several skin conditions, including wounds. Even if in vitro and in vivo studies have highlighted the pro-healing effects of some probiotic bacteria, the underlying mechanisms are still not fully defined. The current investigation aimed to determine the re-epithelialization potential of the soluble fraction from lysate of seven different probiotic strains belonging to different genera (i.e., *Streptococcus*, *Lactobacillus*, and *Bifidobacterium*) on in vitro physically wounded HaCaT monolayer model. The results suggested that the soluble fraction of *S. thermophilus,* *L. plantarum*, and *L. acidophilus* promoted the re-epithelialization of scratched HaCaT monolayers, whereas those from *B. longum*, *B. infantis*, and *B. breve* significantly inhibited the process. On the other hand, *L. bulgaricus* showed no significant effect on in vitro wound repair. The mechanisms underlying the pro- or anti-healing properties of selected bacterial strains strictly and positively correlated with their ability to modulate nitric oxide synthase 2 (NOS2) expression and activity. Accordingly, the pre-treatment with aminoguanidine (AG), a specific inhibitor of NOS2 activity, abrogated the pro-healing effects of *S. thermophilus, L. plantarum*, and *L. acidophilus*.

## 1. Introduction

According to the Food and Agriculture Organisation of the United Nations (FAO) and the World Health Organisation (WHO), probiotics are defined as live microorganisms, which, when administered in adequate amounts, confer a health benefit on the host [1,2]. The most widely used probiotics are species from the genera *Lactobacillus*, *Bifidobacterium*, *Saccharomyces*, *Enterococcus*, *Streptococcus*, *Pediococcus, Leuconostoc*, *Bacillus*, *Escherichia coli* [3]. Oral administration of probiotics has shown to exert positive effects on intestinal and extra-intestinal disorders, including skin diseases [4,5,6,7]. Anyway, there is a growing body of research involving the use of a topical application of probiotics in dermatology with benefits in atopic dermatitis, acne, seborrheic dermatitis, and wound healing [4,7,8,9,10]. In this context, for some years, our group has been focusing the attention on studies aimed to investigate, in vitro and in vivo, the effects of selected probiotic strains on skin. So, we showed evidence of a significant increase in skin ceramide levels in healthy subjects or atopic dermatitis patients after topical treatment with a cream containing an *S. thermophilus* lysate [11,12,13]. The presence of high levels of bacterial sphingomyelinase activity was shown to be responsible for the observed increase of skin ceramide levels, thus leading to an improvement in barrier function and maintenance of *stratum corneum* flexibility. In addition, with the aim to investigate the anti-inflammatory properties and immunomodulatory activities of probiotics, we showed the ability of a *Bifidobacterium infantis* lysate to prevent the abnormal apoptosis of HaCaT cells induced by soluble factors (IFN-γ and CD95 ligand) released by human T-lymphocytes activated in vitro with anti-CD3/CD28 monoclonal antibodies or the mitogen PHA (phytohemagglutinin) [14]. More recently, we described our experience on fractional CO_2_ laser resurfacing providing evidence on a new post-operative topical treatment with an experimental cream containing an *S. thermophilus* lysate able to modulate the inflammatory reaction associated with laser treatment [15]. The topical application of probiotics or their lysates/extracts on skin wounds has been shown to promote healing through the inhibition of the growth of pathogenic bacteria, the regulation of local inflammatory response and the interaction with epidermis cells [16,17,18,19]. The enhancement of tight-junction barrier function in human primary keratinocytes was observed after treatment with *Lactobacillus* and *Bifidobacterium* lysates even if the involved mechanisms depended on the bacterial strain [20]. Moreover, live *L. rhamnosus* GG and its lysate protected primary human keratinocytes against the effects of *S. aureus*, inhibiting the pathogen growth, reducing the bacterial adhesion and increasing the viability of the infected keratinocytes, thus supporting the protective abilities of probiotics towards skin pathogens [21]. *L. rhamnosus* GG lysate also increased re-epithelialization of keratinocyte scratch assay by promoting keratinocyte migration and proliferation through a mechanism which potentially involved increasing expression of the chemokine, CXCL2 and its receptor, CXCR2 [22]. A number of in vivo studies also showed that selected probiotic bacteria could positively affect the wound healing process by topical administration [17]. Topical bacteriotherapy with *L. plantarum* was reported to improve chronic ulcers of non-diabetic and diabetic patients by decreasing bacterial load, neutrophils, apoptotic and necrotic cells, and reducing the area of the lesions through the regulation of interleukin-8 [23]. Probiotics have also been shown to improve wound healing in burn patients and to prevent the risk of infection and bacterial load in the human second- and third-degree burns while promoting granulation tissue [18]. Application of *L. plantarum* reduced *Pseudomonas aeruginosa* burn infections in a mouse model [24]. The use of probiotic formulations would thus represent a valid alternative approach to overcome the existing problems of actual wound therapy approaches, including the high costs, the long manufacturing times, and the increase in antibiotic resistance. However, further studies are needed both to identify probiotics or any combinations of them in terms of therapeutic efficacy and to fully define the underlying mechanisms. In this regard, it seems quite surprising that, according to our knowledge, there is no literature data about possible involvement in the pro-healing mechanisms activated by some probiotics, of nitric oxide (NO), one of the most important players in the regulation of the wound repair process [25,26,27,28]. The levels of NO metabolites were shown to correlate with the healing trajectory indicating the tendency of recovery or exacerbation [29]. The application of exogenous gaseous NO or the nitric oxide synthase 2 (NOS2) stimulator [30], the transfer of the NOS2 gene [31], and the systemic supply of the NOS substrate, i.e., arginine [32], the employment of NO donor systems [33] are all approaches capable of elevating the local NO concentration, and consequently, promoting wound recovery. On the other hand, a blockade or knockout of NOS2 impaired wound healing [34,35,36,37]. Given the crucial role played by NOS2/NO system in the wound repair process, in the present study, after comparing the ability of the soluble fraction from lysates of seven different probiotic strains to affect the re-epithelialization process *in vitro*, we investigated the involvement of NOS2 expression and activity in the mechanisms underlying the probiotic effects in the model of scratched keratinocyte monolayer.

## 2. Materials and Methods 

### 2.1. Preparation of Bacterial Samples for Cell Treatments

*B. longum* BL-04, *B. infantis* Bi-07, *B. breve* BB-03, *S. thermophilus* St-21, *L. bulgaricus* TR-160, *L. plantarum* Lp-115, and *L. acidophilus* LA-14 in a pure lyophilized form were kindly provided by Prof. Claudio De Simone, MD. For bacterial sample preparations, stocks of 10^8^ CFU/g of each lyophilized probiotic resuspended in Phosphate Buffered Saline (PBS, Euro Clone, West York, UK) were centrifuged at 8600 × *g*, washed twice, resuspended in 10 mL of PBS and sonicated (30 min, alternating 10 s of sonication and 10 s of pause) using a Vibracell sonicator (Sonic and Materials, Danbury, CT, USA). Bacterial cell disruption was verified by measuring the absorbance of the sample at 590 nm (Eppendorf Hamburg, Germany) before and after every sonication step. The samples were then centrifuged at 17,949 × *g* and the supernatants filtered using a 0.22-μm-pore filter (Corning Incorporated, Corning, NY, USA) to remove any whole bacteria remaining. Total protein content was determined by DC Protein Assay (BioRad, Hercules, CA) using bovine serum albumin (BSA, Sigma Aldrich, Saint Louis, MO, USA) as standard. Based on our experience, for in vitro experiments, the bacterial soluble fraction were added to cell cultures for different time intervals at 50 μg protein/mL as final concentration. 

### 2.2. Cell Lines and Culture Conditions 

The spontaneously immortalized human keratinocyte HaCaT cell line was purchased from Cell Lines Service GmbH (Eppelheim, Germany) [38]. HaCaT cells were cultured in DMEM supplemented with 10% (*v*/*v*) fetal calf serum (FCS), 2 mM L-glutamine, 100 U/mL penicillin and 100 μg/mL streptomycin (Euro Clone, West York, UK). Culture conditions were kept constant at 37 °C in a 5% CO_2_ humidified atmosphere. Under these culture conditions, HaCaT cells remain proliferative and undifferentiated. After reaching 80% confluence, cells were seeded, as below specified, into a sterile tissue culture 6-well plate or 12-well plate (Becton Dickinson, San Jose, CA, USA) at 18,000 cells/cm^2^. To evaluate the effect of the soluble fraction of bacterial lysate on cell viability and proliferation, the cells were cultured in 12-well plate with bacterial samples at 50 µg protein/mL for different time intervals (20–48 h) after which were washed with PBS, centrifuged for 10 min at 400× *g* and the pellets incubated with a 0.04% Trypan blue (Euro Clone, West York, UK) solution for 5 min to analyse cell number and viability. Not-treated cells were also analyzed and served as controls. Cells were transferred to a Bürker counting chamber and counted by microscopy (Eclipse 50i, Nikon Corporation, Japan). At all the time intervals of incubation with bacterial soluble fraction at 50 µg protein/mL, no significant influence on the cell viability or basal proliferation level compared to the untreated cells was registered, being in all experimental conditions cell viability >90%. 

### 2.3. In Vitro Wound Healing Model

As previously described [39], for the in vitro wound healing assay, HaCaT cells were cultured in 6-well microplates under normal culture conditions to reach ~90% confluence, then DMEM was removed from the wells and cell monolayers were scratched using a 200 µL pipet tip to create a uniform cell-free wound area with reproducible width of wounding. Debris was removed from the culture by gently washing with sterile PBS. Cell cultures were then incubated with fresh medium at 37°C in a 5% CO_2_ humidified atmosphere in the presence or absence of bacterial soluble fraction at 50 μg protein/mL as final concentration. Where indicated, cells were pre-treated for 15 min with 20 μM aminoguanidine (AG), a selective NOS2 inhibitor [40] (Sigma Aldrich, St. Louis, MO, USA). Cell migration was monitored by microscopy using an inverted light microscope (Eclipse TS 100, Nikon) and images were captured (10× magnification) at different time points after the injury (0–45 h). The experiments were conducted in duplicate with at least three fields evaluated for each condition. To calculating the % wound closure, the images acquired were analyzed quantitatively using the TScratch software [41]. Quantification of relative wound closure was performed according to the equation:

(1) %Relative Re-epithelialization=[%scratched area at T0−%scratched area at Tn](×100)[%scratched area at T0]

where Tn is a specific time point (h) after the scratching.

### 2.4. Western Blot Analysis of NOS2 Expression

For Western blot analyses, scratched monolayers of untreated cells and bacterial soluble fraction-treated cells for 28 h were harvested, washed in PBS and lysed in RIPA buffer (Merck KGaA, Darmstadt, Germany) containing a protease inhibitor mixture (carboxypeptidase inhibitor, 5 μg/mL trypsin inhibitor, 1 mM PMSF, 10 μg/mL leupeptin, 10 μg/mL aprotinin, 10 μg/mL pepstatin) (Sigma Aldrich, St. Louis, MO, USA). The samples were assayed for protein content with DC Protein Assay (BioRad, Hercules, CA, USA) using BSA as standard. 25 μg of proteins was mixed with sample buffer, boiled for 5 min at 100 °C and separated by 10% SDS-polyacrylamide gel electrophoresis. Proteins were transferred onto 0.45 µm nitrocellulose membrane sheets (BioRad) for 1 h at 4 °C at 70V using a Mini Trans-Blot Cell apparatus (BioRad). Membranes were blocked with 5% not-fat dry milk for 1 h at room temperature and then incubated overnight at 4 °C with rabbit polyclonal antibody anti-NOS2 1:500 (Cell Signaling Technology, CA, USA) or with goat anti-β-actin antibody 1:1000 (Santa Cruz Biotechnology, Santa Cruz, CA, USA). Horseradish peroxidase (HRP)-conjugated goat anti-rabbit IgG secondary antibody was used at 1:5000 for anti-NOS2 antibody and horseradish peroxidase (HRP)-conjugated rabbit anti-goat IgG secondary antibody at 1:5000 for anti-β-actin antibody (Millipore EMD, Darmstadt, Germany). Immuno-reactive bands were visualized by enhanced chemiluminescence (ECL, Amersham Pharmacia Biotech), according to the manufacturer’s instructions. Band relative densities were determined using a chemiluminescence documentation system ALLIANCE (UVITEC, Cambridge UK), and values were given as relative units. Immunoblot data were normalized to β-actin protein levels.

### 2.5. Nitrite Level Assay

The enzymatic activity of NOS2 was evaluated by measuring nitrite levels using nitrate reductase and Griess reaction through a colorimetric assay (Nitrite Assay kit-Sigma-Aldrich Co., Milan, Italy). Supernatants (80 μL) of the scratched monolayer of untreated cells and bacterial soluble fraction-treated cells for 28 h were applied to a microtiter plate well for nitrite analysis according to the manufacturer’s instructions. The absorbance was measured by spectrophotometric reading at 550 nm using a microplate reader (Bio-Rad Hercules). The nitrite content of each sample was evaluated with a standard curve obtained by linear regression made with sodium nitrite and expressed in μg/mL. Each sample was assayed in duplicate.

### 2.6. Statistical Analysis

Data were analyzed using Prism 6.0 GraphPad Software, San Diego, Ca. Results are expressed as means of three experiments in duplicate ± SEM. For the comparison of groups, a one or two-way ANOVA test followed by Bonferroni or Dunnett post hoc test were used, as specified below. The statistical correlation was calculated through Pearson’s test. Results were considered significant if *p* < 0.05. For statistical analysis of data sets * or ^#^ as *p* < 0.05, ** or ^##^ as *p* < 0.01, *** or ^###^ as *p* < 0.001, and **** or ^####^ as *p* < 0.0001, were used throughout the manuscript.

## 3. Results

### 3.1. Effects of the Soluble Fraction from Bacterial Lysates on Scratched HaCaT Monolayer Wound Healing

**Figure 1:**
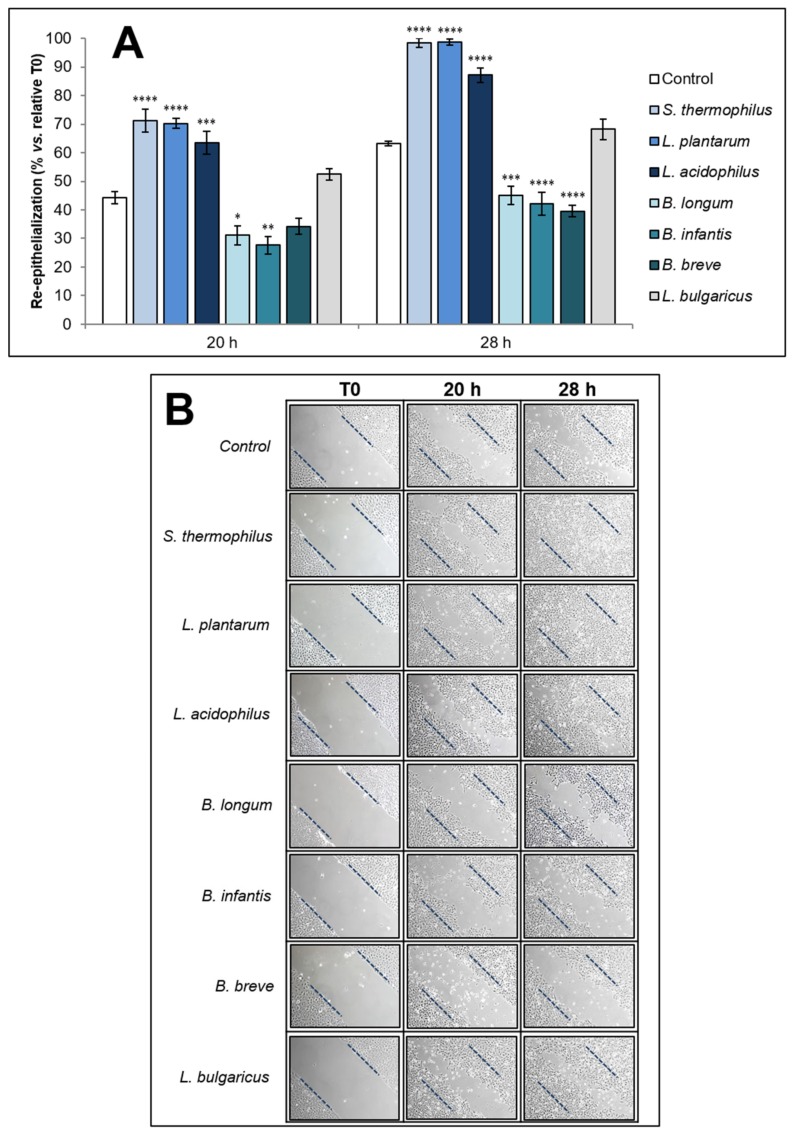
Effect of the soluble fraction of bacterial lysates on re-epithelialization of HaCaT cell line scratched monolayers at different time points. (**A**) Effect of bacterial samples (50 µg/mL) on the wound closure rate (% vs. relative T0) of scratched monolayers at 20 and 28 h. Data are expressed as mean ± SEM of three independent experiments in duplicate. For comparative analysis of groups of data, a two-way analysis of variance (ANOVA) with post hoc Dunnett test was used. * *p* < 0.05, ** *p* < 0.01, *** *p* < 0.001, **** *p* < 0.0001 vs. control (untreated). (**B**) Representative images of HaCaT monolayer re-epithelialization in the absence (control) or presence of bacterial samples (50 µg/mL) at 20 and 28 h after injury (10× magnification).

The ability of the soluble fraction from bacterial lysates to influence re-epithelialization was assessed on an in vitro artificial wound model. The rate of scratched monolayer closure in the absence or presence of 50 μg/mL of each bacterial sample was evaluated by observing the re-population of the area between the wound edges at different time points after the lesion. To quantitatively analyze the effects of the bacterial soluble fraction on the closure of the wounded area, images obtained by inverted light microscope were acquired at different time points after scratching and converted to % closure by using a mathematical system calculating automatically the portion of the area occupied by the cells. In all experiments, the scratched monolayers of control cells (untreated) were closed at 36–42 h. The percentages of re-epithelialization at 20 and 28 h in the presence or absence of bacterial samples were compared to the untreated monolayers. The results expressed as relative re-epithelialization percentages (mean ± SEM from three independent experiments in duplicate), as well as representative images from microscopic observations of scratched monolayers, are shown in Figure 1A,B, respectively. 

The treatment with the soluble fraction from lysates of *S. thermophilus*, *L. plantarum* or *L. acidophilus* significantly accelerated the rate of monolayer repair process respect to untreated control at both 20 and 28 h from injury. On the contrary, the treatment with *B. longum*, *B. infantis*, and *B. breve* samples significantly delayed monolayer repair process compared to untreated control being the effects more statistically relevant at 28 h. On the other hand, *L. bulgaricus* sample didn’t appear to significantly influence the wound closure rate at both observation times when compared to control. 

### 3.2. NOS2 Expression and Nitrite Levels

**Figure 2:**
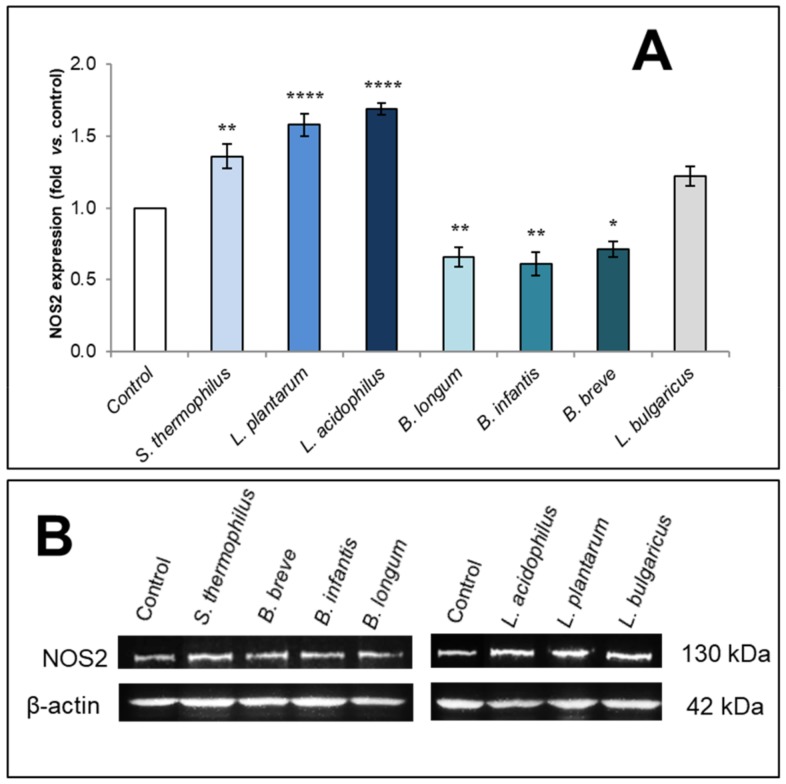
Influence of the soluble fraction from probiotic lysates on NOS2 protein levels in scratched HaCaT monolayers. Immunoblotting assay for NOS2 was performed on scratched monolayers treated for 28 h with 50 µg/mL of probiotic soluble fraction. Following densitometric analysis, obtained values were normalized vs. β-actin and compared with untreated controls. (**A**) Results of densitometry expressed as ratio NOS2/β-actin (fold vs. control). Data are from three independent experiments in duplicate and values are expressed as mean ± SEM. For comparative analysis of data, a one-way analysis of variance (ANOVA) with post hoc Dunnett test was used * *p* < 0.05, *** *p* < 0.001, **** *p* < 0.0001 vs. control (untreated). (**B**) A representative image of immunoblotting for NOS2 is shown.

To investigate the potential involvement of NOS2 in the above-reported effects of the soluble fraction from bacterial lysates on HaCaT monolayer repair process, we analyzed by western blot the NOS2 protein levels in monolayers after 28 h from scratch in the absence or presence of bacterial samples at 50 µg/mL. The values obtained following densitometric analysis of NOS2 bands were normalized versus β-actin. Data expressed as mean ± SEM from three independent experiments in duplicate together with a representative image of immunoblotting for NOS2 are shown in Figure 2A,B, respectively. 

In line with their ability to accelerate monolayer re-epithelialization, the results indicated that the treatment with the soluble fraction from *S. thermophilus*, *L. plantarum*, and *L. acidophilus* lysates led to a marked and significant upregulation of NOS2 protein expression vs. control cells. The most relevant effect could be observed with *L. acidophilus* and *L. plantarum* (*p* < 0.0001 vs. control). *S. thermophilus* sample was also able to significantly increase NOS2 protein expression level even if with statistically lower efficacy (*p* < 0.01). On the other hand, *L. bulgaricus* sample, which did not significantly affect monolayer wound repair, was also unable to modulate NOS2 expression compared to control. Of note, HaCaT monolayer exposed to *B. longum*, *B. infantis,* and *B. breve* samples, all strains able to inhibit the wound closure rate, showed significantly lower NOS2 protein levels compared to untreated condition (*p* < 0.01 with *B. longum* or *B. infantis*; *p* < 0.05 for *B. breve)*.

**Figure 3:**
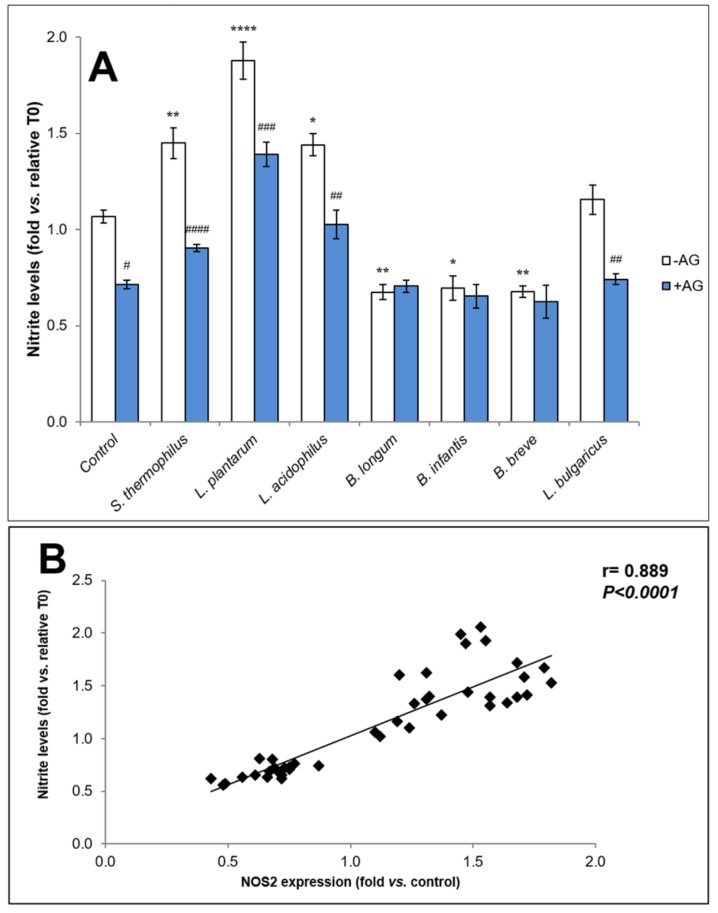
Nitrite levels in HaCaT scratched monolayers’ medium in the presence or absence of NOS2 inhibitor AG. (**A**) HaCaT scratched monolayers were incubated with or without 20 µM AG for 15 min before bacterial soluble fraction treatment. After 28 h, nitrite levels in the culture medium were analyzed by Griess reagent. Data shown are expressed as mean ± SEM of three independent experiments in duplicate. For comparative analysis of groups of data, a two-way analysis of variance (ANOVA) with post hoc Bonferroni test was used. * *p* < 0.05, ** *p* < 0.01, **** *p* < 0.0001 vs. control (untreated). ^#^ *p* < 0.05, ^##^ *p* < 0.01, ^###^ *p* < 0.001, ^####^ *p* < 0.0001 vs. each relative culture without AG. (**B**) Pearson’s test analysis of the correlation between NOS2 expression (fold vs. control) and nitrite levels (fold vs. relative T0).

The ability of the bacterial samples to regulate NOS2 activity was analyzed by measuring nitrite levels in supernatants of scratched HaCaT monolayers pre-treated with or without the selective NOS2 inhibitor, AG (Figure 3A). Pre-treatment with AG prevented the increase of nitrite generation due to NOS activity induced by the scratch of control monolayer (*p* < 0.05), thus confirming our previous findings [39]. The treatment with *S. thermophilus, L. plantarum,* and *L. acidophilus* samples, able to up-modulate NOS2 expression, induced a significant increase of nitrite levels in culture medium *versus* control, even if at different extent (*S. thermophilus*, *p* < 0.01; *L. plantarum*, *p* < 0.0001; *L. acidophilus p* < 0.05). Of note, the stimulatory effect of *S. thermophilus*, *L. acidophilus*, and *L. plantarum* samples on nitrite generation was totally o partially prevented by pre-treatment with AG, thus supporting the ability of these probiotics to induce NOS2 expression and activity in our experimental conditions. On the contrary, *B. longum*, *B. infantis*, or *B. breve* samples induced a significant decrease of nitrite levels respect to control, confirming their inhibitory effect on NOS2 expression at a similar extent (*B. longum p* < 0.01; *B. infantis p* < 0.05; *B. breve p* < 0.01). In accordance with the results of experiments on NOS2 expression, pre-treatment with AG did not significantly influence nitrite levels in cell cultures treated with soluble fraction from *B. longum*, *B. infantis*, and *B. breve* lysates as compared to the monolayers treated with bacterial samples alone. The treatment with *L. bulgaricus* sample did not significantly influence nitrite levels compared to untreated cultures, in line with the above results on NOS2 expression and, like control condition, AG pre-treatment significantly decreased nitrite amounts as compared to the relative sample without AG. Using Pearson’s test, a significant linear positive correlation resulted between NOS2 expression (fold vs. control) and nitrite level (fold vs. relative T0) (*r = 0.889*; *p* < 0.0001) (Figure 3B). 

**Figure 4:**
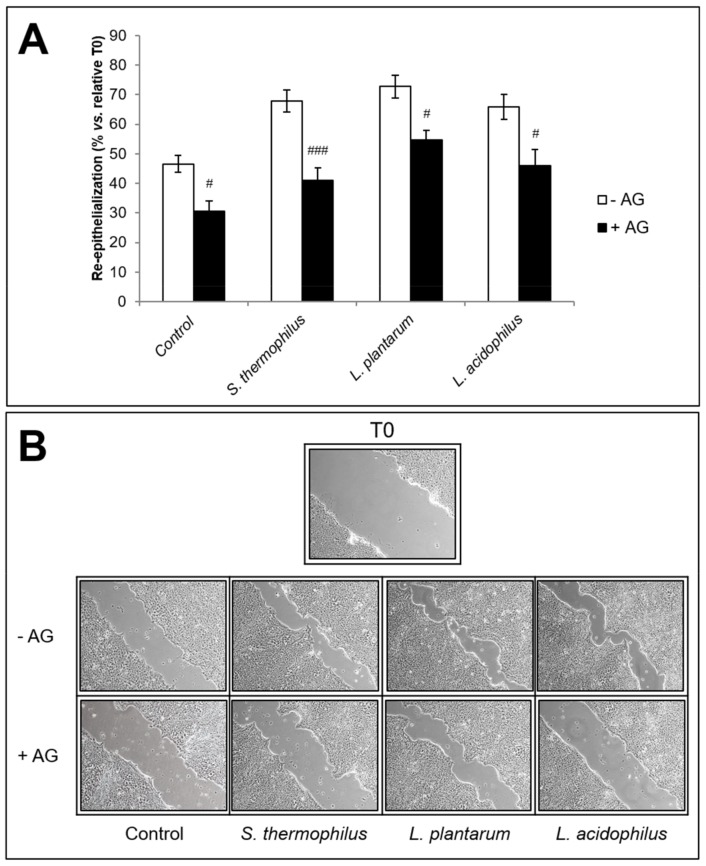
Effect of NOS2 inhibitor AG on the re-epithelialization of HaCaT scratched monolayers induced by *S. thermophilus*, *L. plantarum*, and *L. acidophilus*. (**A**) Effect of 15 min pre-treatment with 20 µM AG on the relative wound closure rate (%) of scratched monolayers with or without the indicated probiotic lysate-soluble fraction at 50 µg/mL for 20 h. Data are expressed as mean ± SEM of three independent experiments in duplicate. For comparative analysis of groups of data, a two-way analysis of variance (ANOVA) with post hoc Bonferroni test was used. ^#^ *p* < 0.05, ^###^ *p* < 0.001 vs. each relative culture without AG. (**B**) Representative images of HaCaT cell line scratched monolayers pre-treated with or without 20 µM AG for 15 min and then with *S. thermophilus, L. plantarum* or *L. acidophilus* samples at 50 µg/mL for 20 h (10× magnification).

The effect of NOS2-inhibitor AG on the rate of wound closure was also evaluated on the ability of *S. thermophilus, L. plantarum*, and *L. acidophilus* lysate-derived soluble fraction to accelerate wound closure process *in vitro*. Results, expressed as mean ± SEM of three experiments in duplicate, are relative to the % re-epithelialization at 20 h vs. relative T0 from monolayer scratching in the presence or absence of bacterial samples at 50 µg/mL (Figure 4A). Representative images from microscopic observations are also shown (Figure 4B). As expected, in accordance with our previous findings [39], the pre-treatment with AG strongly affected the physiological repair of control monolayer and significantly prevented the stimulating effect of all these bacterial samples in terms of monolayer repair rate expressed as % re-epithelialization vs. relative T0. 

**Figure 5:**
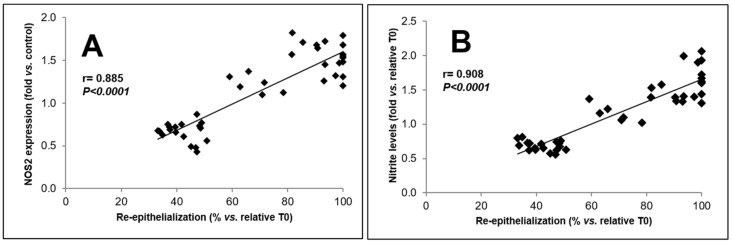
Analysis of correlation between NOS2 expression, nitrite levels, and wound closure rate. (**A**) Pearson’s test analysis of the correlation between NOS2 expression (fold vs. control) and wound closure rate expressed as % re-epithelialization vs. relative T0. (**B**) Pearson’s test analysis of correlation between nitrite levels (fold vs. relative T0) and wound closure rate expressed as % re-epithelialization vs. relative T0.

To verify the statistical relationship between the percentages of wound closure, NOS2 expression, and nitrite levels, Pearson’s correlation test was used. Statistical analysis revealed a positive, strong, statistically significant correlation between NOS2 protein levels (fold vs. control) and wound closure (% re-epithelialization vs. relative T0) (*r* = 0.885; *p* < 0.0001) (Figure 5A). Similarly, Pearson’s test revealed a strong correlation also between nitrite levels (fold vs. relative T0) and wound closure (% re-epithelialization vs. relative T0) (*r* = 0.908; *p* < 0.0001) (Figure 5B). Taken together these results, confirming the involvement of NO in the wound repair process, strongly suggest that up-modulation of NOS2 expression and activity plays a crucial role in the re-epithelialization process elicited by the soluble fraction from probiotic lysates.

## 4. Discussion

**Figure 6:**
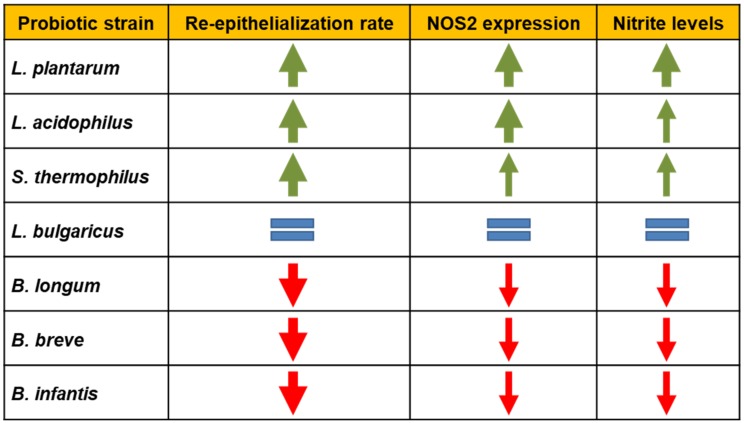
Effects of the soluble fraction from lysates of the used probiotic strains on scratched HaCaT monolayers: Overview of the re-epithelialization potential of the used probiotic strains as well as their ability to influence NOS2 protein expression and activity.

Considering the crucial barrier function of the epidermis, when an injury occurs, it is necessary to re-establish tissue integrity as fast and efficiently as possible, through the re-epithelialization process. Keratinocyte proliferation and migration represent an essential step in the re-epithelialization process during wound healing [42]. In the present study, we compared the influence exerted by the soluble fraction from lysates of seven different probiotic strains at the same experimental conditions on the in vitro wound healing model. Samples of *S. thermophilus, L. plantarum*, and *L. acidophilus*, promoted the re-epithelialization of scratched HaCat monolayers. In contrast, the soluble fraction from *B. longum, B. infantis*, and *B. breve* lysates inhibited the monolayer repair process, whereas *L. bulgaricus* had no effect. Given the key role of the NO generation in the wound healing process and with the aim to identify the mechanisms mediating the effects of the probiotic samples observed in our experiments, we examined the possible involvement of the NOS2/NO system in the ability of probiotics to influence wound healing in vitro. The acceleration of re-epithelialization induced by *S. thermophilus, L. plantarum*, and *L. acidophilus*, was associated with an increase of NOS2 expression and activity, as demonstrated by immunoblotting data and nitrite level assay. Of note, the ability of the soluble fraction from *S. thermophilus, L. plantarum*, and *L. acidophilus* lysates to accelerate re-epithelialization was abrogated by the inhibition of NOS2 by AG, thus confirming that the beneficial effect of these probiotics may be mediated by NOS2/NO pathway. On the other hand, *B. infantis*, *B. breve*, and *B. longum*, significantly downmodulated NOS2 expression and activity in scratched HaCat monolayers. *L. bulgaricus*, also in this context, did show no effect. Figure 6, with the aid of arrows, summarizes the effects of all used probiotics on keratinocyte re-epithelialization, NOS2 expression, and nitrite levels. Taken together these results strongly suggest that the pro- or anti-healing properties of probiotics used are strictly strain-dependent and associated with their ability to up- or down-modulate NOS2 expression and activity, as also supported by the Pearson’s correlation analyses.

The results of a recent meta-analysis from controlled in vivo studies testing the efficacy of probiotics for skin lesions in animal models strongly support that topical probiotic administration is an effective pharmacological treatment for cutaneous wounds [43]. The new perspective and therapeutic potential of beneficial probiotic species as an alternative and safe approach for the treatment of patients affected by cutaneous disorders, including non-healing wounds, has been recently reviewed [7,17]. Our data extend the spectrum of the mechanisms underlying the effects of specific probiotics on the re-epithelialization process and may further justify their use in the topical treatment of skin wounds. Even if further in vivo studies should be conducted to confirm their potential therapeutic use, it will be of interest to firstly verify the reproducibility of the observed effects on HaCat cell line, also on primary keratinocytes, as well as other cell types involved in skin wound healing process (i.e., fibroblasts, endothelial cells), focusing on the biomolecular mechanism behind the observed effects. In this regard, considering the role attributed to the pro-inflammatory cytokines IL-6 and IL-8 in the tissue repair process [44,45,46] as well their association with NOS2 expression in different cellular models [47,48,49,50,51], our preliminary results (Figure S1, Supplementary Materials) show that *S. thermophilus*, *L. plantarum*, and *L. acidophilus* samples, able to positively modulate NOS2/NO system and stimulate scratched monolayer repair, significantly increased the secretion of these two cytokines by HaCat; conversely *B. infantis*, *B. breve*, and *B. longum,* which caused a reduction of NOS2 expression and activity, also led to decreased levels of IL-6 and IL-8. *L. bulgaricus*, also in this context, did show no effect. IL-6 and IL-8 are reported to be involved in the phosphatidylinositol 3 kinase (PI3K)/protein kinase B (Akt) pathway [52]; interestingly *Lactobacillus rhamnosus* and *Lactobacillus reuteri* lysates promoted wound healing process via the PI3K/Akt pathway in a reconstructed human epidermis model and gingiva mesenchymal stem cells, respectively [53,54]. Based on these observations, the involvement of the PI3K/Akt pathway in the acceleration of the in vitro wound healing process stimulated by *S. thermophilus*, *L. plantarum*, and *L. acidophilus* samples could be also examined.

Finally, the accurate characterization of the subcellular components responsible for the observed probiotic pro-healing effects is another essential aspect. In this context, our group is working to identifying, through cell extraction and purification techniques, the factor or the cocktail of factors from the three identified probiotic strains able to accelerate the epithelial repair process.

Moreover, the standardization of the dose and appropriate delivery represent crucial factors for probiotic efficacy. In addition, our findings strongly support that the choice of the probiotic strains should also be considered of crucial importance, as the effects of these bacteria are highly strain-specific. Finally, in the light of a number of recent scientific reports [55,56,57,58,59,60,61], changes in the manufacturing processes, equipment, or facilities, which could result in detrimental differences in the final probiotic product, should be strictly controlled in terms of safety and efficacy.

## Supplementary Materials

The following is available online at https://www.mdpi.com/2218-273X/9/12/756/s1, Figure S1: IL-6 and IL-8 levels in the culture medium of HaCaT scratched monolayers treated with the soluble fraction from probiotic lysates.

## Abbreviations

NOS2	Nitric oxide synthase 2
NO	Nitric oxide
AG	Aminoguanidine

## References

[1] Food and Agriculture Organization of the United Nations/World Health Organization FAO/WHO (2001). Health and Nutritional Properties of Probiotics in Food including Powder Milk with Live Lactic Acid Bacteria. http://www.who.int/foodsafety/publications/fs_management/en/probiotics.pdf.

[2] Food and Agriculture Organization of the United Nations/World Health Organization FAO/WHO (2002). Guidelines for the Evaluation of Probiotics in Food.

[3] Fijan S. (2014). Microorganisms with Claimed Probiotic Properties: An Overview of Recent Literature. Int. J. Environ. Res. Public Health.

[4] Kiousi D.E., Karapetsas A., Karolidou K., Panayiotidis M.I., Pappa A., Galanis A. (2019). Probiotics in Extraintestinal Diseases: Current Trends and New Directions. Nutrients.

[5] Nole K.L.B., Yim E., Keri J.E. (2014). Probiotics and prebiotics in dermatology. J. Am. Acad. Dermatol..

[6] Tsai Y.L., Lin T.L., Chang C.J., Wu T.R., Lai W.F., Lu C.C., Lai H.C. (2019). Probiotics, prebiotics and amelioration of diseases. J. Biomed. Sci..

[7] Yu Y., Dunaway S., Champer J., Kim J., Alikhan A. (2019). Changing our microbiome: Probiotics in dermatology. Br. J. Dermatol..

[8] Cinque B., La Torre C., Melchiorre E., Marchesani G., Zoccali G., Palumbo P., Di Marzio L., Masci A., Mosca L., Mastromarino P. (2011). Use of Probiotics for Dermal Applications. Microbiol. Monogr..

[9] Cinque B., Palumbo P., La Torre C., Melchiorre E., Corridoni D., Miconi G., Di Marzio L., Cifone M.G., Giuliani M. (2010). Probiotics in aging skin. Textb. Aging Skin.

[10] Notay M., Foolad N., Vaughn A.R., Sivamani R.K. (2017). Probiotics, Prebiotics, and Synbiotics for the Treatment and Prevention of Adult Dermatological Diseases. Am. J. Clin. Dermatol..

[11] Di Marzio L., Centi C., Cinque B., Masci S., Giuliani M., Arcieri A., Zicari L., De Simone C., Cifone M.G. (2003). Effect of the lactic acid bacterium Streptococcus thermophilus on stratum corneum ceramide levels and signs and symptoms of atopic dermatitis patients. Exp. Dermatol..

[12] Di Marzio L., Cinque B., Cupelli F., De Simone C., Cifone M.G., Giuliani M. (2008). Increase of skin-ceramide levels in aged subjects following a short-term topical application of bacterial sphingomyelinase from Streptococcus thermophilus. Int. J. Immunopathol. Pharmacol..

[13] Di Marzio L., Cinque B., De Simone C., Cifone M.G. (1999). Effect of the lactic acid bacterium Streptococcus thermophilus on ceramide levels in human keratinocytes in vitro and stratum corneum in vivo. J. Investig. Dermatol..

[14] Cinque B., Di Marzio L., Della Riccia D.N., Bizzini F., Giuliani M., Fanini D., De Simone C., Cifone M.G. (2006). Effect of Bifidobacterium infantis on interferon-gamma-induced keratinocyte apoptosis: A potential therapeutic approach to skin immune abnormalities. Int. J. Immunopathol. Pharmacol..

[15] Zoccali G., Cinque B., La Torre C., Lombardi F., Palumbo P., Romano L., Mattei A., Orsini G., Cifone M.G., Giuliani M. (2016). Improving the outcome of fractional CO_2_ laser resurfacing using a probiotic skin cream: Preliminary clinical evaluation. Laser Med. Sci..

[16] Huseini H.F., Rahimzadeh G., Fazeli M.R., Mehrazma M., Salehi M. (2012). Evaluation of wound healing activities of kefir products. Burns.

[17] Lukic J., Chen V., Strahinic I., Begovic J., Lev-Tov H., Davis S.C., Tomic-Canic M., Pastar I. (2017). Probiotics or pro-healers: The role of beneficial bacteria in tissue repair. Wound Repair Regen..

[18] Peral M.C., Martinez M.A.H., Valdez J.C. (2009). Bacteriotherapy with Lactobacillus plantarum in burns. Int. Wound J..

[19] Wong V.W., Martindale R.G., Longaker M.T., Gurtner G.C. (2013). From Germ Theory to Germ Therapy: Skin Microbiota, Chronic Wounds, and Probiotics. Plast. Reconstr. Surg..

[20] Sultan R., McBain A.J., O’Neill C.A. (2013). Strain-Dependent Augmentation of Tight-Junction Barrier Function in Human Primary Epidermal Keratinocytes by Lactobacillus and Bifidobacterium Lysates. Appl. Environ. Microbiol..

[21] Mohammedsaeed W., McBain A.J., Cruickshank S.M., O’Neill C.A. (2014). Lactobacillus rhamnosus GG inhibits the toxic effects of Staphylococcus aureus on epidermal keratinocytes. Appl. Environ. Microbiol..

[22] Mohammedsaeed W., Cruickshank S., McBain A.J., O’Neill C.A. (2015). Lactobacillus rhamnosus GG Lysate Increases Re-Epithelialization of Keratinocyte Scratch Assays by Promoting Migration. Sci. Rep..

[23] Peral M.C., Rachid M.M., Gobbato N.M., Martinez M.A.H., Valdez J.C. (2010). Interleukin-8 production by polymorphonuclear leukocytes from patients with chronic infected leg ulcers treated with Lactobacillus plantarum. Clin. Microbiol. Infect..

[24] Valdez J.C., Peral M.C., Rachid M., Santana M., Perdigon G. (2005). Interference of Lactobacillus plantarum with Pseudomonas aeruginosa in vitro and in infected burns: The potential use of probiotics in wound treatment. Clin. Microbiol. Infect..

[25] Abd El-Aleem S.A., Abdelwahab S., Am-Sherief H., Sayed A. (2019). Cellular and physiological upregulation of inducible nitric oxide synthase, arginase, and inducible cyclooxygenase in wound healing. J. Cell. Physiol..

[26] Broughton G., Janis J.E., Attinger C.E. (2006). Wound healing: An overview. Plast. Reconstr. Surg..

[27] Frank S., Kampfer H., Wetzler C., Pfeilschifter J. (2002). Nitric oxide drives skin repair: Novel functions of an established mediator. Kidney Int..

[28] Kumar S., Singh R.K., Bhardwaj T.R. (2017). Therapeutic role of nitric oxide as emerging molecule. Biomed. Pharmacother..

[29] Bernatchez S.F., Menon V., Stoffel J., Walters S.A., Lindroos W.E., Crossland M.C., Shawler L.G., Crossland S.P., Boykin J.V. (2013). Nitric oxide levels in wound fluid may reflect the healing trajectory. Wound Repair Regen..

[30] Shekhter A.B., Serezhenkov V.A., Rudenko T.G., Pekshev A.V., Vanin A.F. (2005). Beneficial effect of gaseous nitric oxide on the healing of skin wounds. Nitric Oxide.

[31] Yamasaki K., Edington H.D., McClosky C., Tzeng E., Lizonova A., Kovesdi I., Steed D.L., Billiar T.R. (1998). Reversal of impaired wound repair in iNOS-deficient mice by topical adenoviral-mediated iNOS gene transfer. J. Clin. Investig..

[32] Shi H.P., Efron D.T., Most D., Tantry U.S., Barbul A. (2000). Supplemental dietary arginine enhances wound healing in normal but not inducible nitric oxide synthase knockout mice. Surgery.

[33] Malone-Povolny M.J., Maloney S.E., Schoenfisch M.H. (2019). Nitric Oxide Therapy for Diabetic Wound Healing. Adv. Healthc. Mater..

[34] Amadeu T.P., Costa A.M. (2006). Nitric oxide synthesis inhibition alters rat cutaneous wound healing. J. Cutan. Pathol..

[35] Frank S., Stallmeyer B., Kampfer H., Kolb N., Pfeilschifter J. (1999). Nitric oxide triggers enhanced induction of vascular endothelial growth factor expression in cultured keratinocytes (HaCaT) and during cutaneous wound repair. FASEB J..

[36] Kitano T., Yamada H., Kida M., Okada Y., Saika S., Yoshida M. (2017). Impaired Healing of a Cutaneous Wound in an Inducible Nitric Oxide Synthase-Knockout Mouse. Dermatol. Res. Pract..

[37] Stallmeyer B., Kampfer H., Kolb N., Pfeilschifter J., Frank S. (1999). The function of nitric oxide in wound repair: Inhibition of inducible nitric oxide-synthase severely impairs wound reepithelialization. J. Investig. Dermatol..

[38] Boukamp P., Petrussevska R.T., Breitkreutz D., Hornung J., Markham A., Fusenig N.E. (1988). Normal keratinization in a spontaneously immortalized aneuploid human keratinocyte cell line. J. Cell Biol..

[39] La Torre C., Cinque B., Lombardi F., Miconi G., Palumbo P., Evtoski Z., Placidi G., Fanini D., Cimini A.M., Benedetti E. (2016). Nitric Oxide Chemical Donor Affects the Early Phases of In Vitro Wound Healing Process. J. Cell. Physiol..

[40] Misko T.P., Moore W.M., Kasten T.P., Nickols G.A., Corbett J.A., Tilton R.G., McDaniel M.L., Williamson J.R., Currie M.G. (1993). Selective inhibition of the inducible nitric oxide synthase by aminoguanidine. Eur. J. Pharmacol..

[41] Geback T., Schulz M.M., Koumoutsakos P., Detmar M. (2009). TScratch: A novel and simple software tool for automated analysis of monolayer wound healing assays. Biotechniques.

[42] Eming S.A., Martin P., Tomic-Canic M. (2014). Wound repair and regeneration: Mechanisms, signaling, and translation. Sci. Transl. Med..

[43] Tsiouris C.G., Kelesi M., Vasilopoulos G., Kalemikerakis I., Papageorgiou E.G. (2017). The efficacy of probiotics as pharmacological treatment of cutaneous wounds: Meta-analysis of animal studies. Eur. J. Pharm. Sci..

[44] Choi J.H., Jun J.H., Kim J.H., Sung H.J., Lee J.H. (2014). Synergistic Effect of Interleukin-6 and Hyaluronic Acid on Cell Migration and ERK Activation in Human Keratinocytes. J. Korean Med. Sci..

[45] Grimstad O., Sandanger O., Ryan L., Otterdal K., Damaas J.K., Pukstad B., Espevik T. (2011). Cellular sources and inducers of cytokines present in acute wound fluid. Wound Repair Regen..

[46] Takada K., Komine-Aizawa S., Hirohata N., Trinh Q.D., Nishina A., Kimura H., Hayakawa S. (2017). Poly I:C induces collective migration of HaCaT keratinocytes via IL-8. BMC Immunol..

[47] Davila-Gonzalez D., Chang J.C., Billiar T.R. (2017). NO and COX2: Dual targeting for aggressive cancers. Proc. Natl. Acad. Sci. USA.

[48] Heinecke J.L., Ridnour L.A., Cheng R.Y.S., Switzer C.H., Lizardo M.M., Khanna C., Glynn S.A., Hussain S.P., Young H.A., Ambs S. (2014). Tumor microenvironment-based feed-forward regulation of NOS_2_ in breast cancer progression. Proc. Natl. Acad. Sci. USA.

[49] Seo J.Y., Yu J.H., Lim J.W., Mukaida N., Kim H. (2009). Nitric oxide-induced IL-8 expression is mediated by NF-kappa B and AP-1 in gastric epithelial ags cells. J. Physiol. Pharmacol..

[50] Soufli I., Toumi R., Rafa H., Touil-Boukoffa C. (2016). Overview of cytokines and nitric oxide involvement in immuno-pathogenesis of inflammatory bowel diseases. World J. Gastrointest. Pharmacol. Ther..

[51] Vuolteenaho K., Koskinen A., Kukkonen M., Nieminen R., Paivarinta U., Moilanen T., Moilanen E. (2009). Leptin Enhances Synthesis of Proinflammatory Mediators in Human Osteoarthritic Cartilage-Mediator Role of NO in Leptin-Induced PGE(2), IL-6, and IL-8 Production. Mediat. Inflamm..

[52] Xie S.B., Chen M., Yan B., He X.F., Chen X.W., Li D.W. (2014). Identification of a Role for the PI3K/AKT/mTOR Signaling Pathway in Innate Immune Cells. PLoS ONE.

[53] Han N.N., Jia L., Su Y.Y., Du J., Guo L.J., Luo Z.H., Liu Y. (2019). Lactobacillus reuteri extracts promoted wound healing via PI3K/AKT/beta-catenin/TGF beta 1 pathway. Stem Cell Res. Ther..

[54] Jung Y.O., Jeong H., Cho Y., Lee E.O., Jang H.W., Kim J., Nam K.T., Lim K.M. (2019). Lysates of a Probiotic, Lactobacillus rhamnosus, Can Improve Skin Barrier Function in a Reconstructed Human Epidermis Model. Int. J. Mol. Sci..

[55] Biagioli M., Laghi L., Carino A., Cipriani S., Distrutti E., Marchiano S., Parolin C., Scarpelli P., Vitali B., Fiorucci S. (2017). Metabolic Variability of a Multispecies Probiotic Preparation Impacts on the Anti-inflammatory Activity. Front. Pharmacol..

[56] Cinque B., La Torre C., Lombardi F., Palumbo P., Evtoski Z., Santini S., Falone S., Cimini A., Amicarelli F., Cifone M.G. (2017). VSL#3 probiotic differently influences IEC-6 intestinal epithelial cell status and function. J. Cell. Physiol..

[57] Cinque B., La Torre C., Lombardi F., Palumbo P., Van der Rest M., Cifone M.G. (2016). Production Conditions Affect the In Vitro Anti-Tumoral Effects of a High Concentration Multi-Strain Probiotic Preparation. PLoS ONE.

[58] Palumbo P., Lombardi F., Cifone M.G., Cinque B. (2019). The Epithelial Barrier Model Shows That the Properties of VSL#3 Depend from Where it is Manufactured. Endocr. Metab. Immune Disord. Drug Targets.

[59] Sanders M.E., Klaenhammer T.R., Ouwehand A.C., Pot B., Johansen E., Heimbach J.T., Marco M.L., Tennila J., Ross R.P., Franz C. (2014). Effects of genetic, processing, or product formulation changes on efficacy and safety of probiotics. Ann. N. Y. Acad. Sci..

[60] Trinchieri V., Laghi L., Vitali B., Parolin C., Giusti I., Capobianco D., Mastromarino P., De Simone C. (2017). Efficacy and Safety of a Multistrain Probiotic Formulation Depends from Manufacturing. Front. Immunol..

[61] Zacarias M.F., Souza T.C., Zaburlin N., Carmona Cara D., Reinheimer J., Nicoli J., Vinderola G. (2017). Influence of Technological Treatments on the Functionality of Bifidobacterium lactis INL1, a Breast Milk-Derived Probiotic. J. Food Sci..

