# Supplementary material for: Soluble Fraction from Lysates of Selected Probiotic Strains Differently Influences Re-Epithelialization of HaCaT Scratched Monolayer Through a Mechanism Involving Nitric Oxide Synthase 2
Source: Biomolecules. 2019 Nov 21;9(12):756. doi: 10.3390/biom9120756 (PMC6995614; doi:10.3390/biom9120756)
Supplement: Supplementary file 1 [file biomolecules-09-00756-s001.pdf]

# Supplementary Materials

Enzyme-linked immunosorbent assay (ELISA) of IL-6 and IL-8.

The IL-6 and IL-8 concentrations in the culture medium were measured using an ELISA kit (Sigma Aldrich, Saint Louis, MO, USA) according to the manufacturer's instructions. Supernatants of the scratched monolayers of untreated cells and bacterial soluble fraction-treated cells for 28 h were collected and centrifuged at 17,949× *g* for 10 min. Cell-free supernatants were then harvested and stored at −20 °C until assay. The absorbance was measured by spectrophotometric reading at 550 nm using a microplate reader (Bio-Rad Hercules, California, USA).

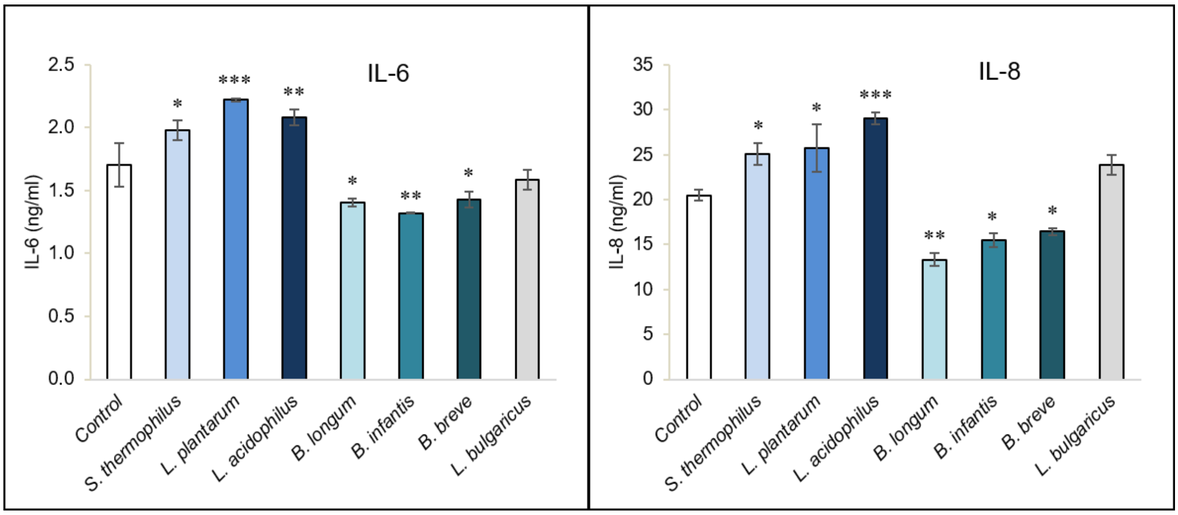


**Figure S1.** IL-6 and IL-8 levels in the culture medium of HaCaT scratched monolayers treated with the soluble fraction from probiotic lysates. HaCaT scratched monolayers were incubated with or without probiotic soluble fraction for 28 h, then IL-6 and IL-8 levels in the culture medium were analyzed by the ELISA kit. Data shown are expressed as mean ± SD of one experiment performed in duplicate. For comparative analysis of groups of data, a one-way analysis of variance (ANOVA) with *post hoc* Dunnett test was used. * $p < 0.05$, ** $p < 0.01$, *** $p < 0.001$ vs. control (untreated).
